# Accumulation of Microvascular Target Organ Damage in Systemic Lupus Erythematosus Patients Is Associated with Increased Cardiovascular Risk

**DOI:** 10.3390/jcm13072140

**Published:** 2024-04-08

**Authors:** Nikolaos Koletsos, Antonios Lazaridis, Areti Triantafyllou, Panagiota Anyfanti, Stamatina Lamprou, Anastasia Stoimeni, Nikolaos G. Papadopoulos, Evaggelia-Evdoxia Koravou, Eugenia Gkaliagkousi

**Affiliations:** 1 3rd Department of Internal Medicine, Papageorgiou General Hospital, Aristotle University of Thessaloniki, 56429 Thessaloniki, Greece; spanbiol@hotmail.com (A.L.); artriant@auth.gr (A.T.); lamproustam@gmail.com (S.L.); anastoim@gmail.com (A.S.); eugalant@yahoo.com (E.G.); 2 Second Medical Department, Hippokration General Hospital, Aristotle University of Thessaloniki, 54642 Thessaloniki, Greece; panyfan@hotmail.com; 3 Rheumatology Department, Papageorgiou General Hospital, 56429 Thessaloniki, Greece; papadopoulosng@gmail.com; 4 BMT Unit, Department of Haematology, G. Papanikolaou General Hospital, 57010 Thessaloniki, Greece; evakikor@gmail.com

**Keywords:** lupus erythematosus, laser speckle, retina, SEVR, QRISK3, microvascular dysfunction, Galectin

## Abstract

**Background**: Systemic lupus erythematosus (SLE) is a prototype autoimmune disease associated with increased cardiovascular (CV) burden. Besides increased arterial stiffness and subclinical atherosclerosis, microvascular dysfunction is considered an important component in the pathophysiology of CV disease. However, there is a lack of data regarding the effect of multiple target organ damage (TOD) on CV health. **Objectives**: This study aimed to evaluate (i) the presence of microvascular changes in SLE in various vascular beds, (ii) the possible associations between the accumulation of microvascular TOD and CV risk and (iii) whether Galectin-3 represents a predictor of combined microvascular TOD. **Methods**: Participants underwent (i) evaluation of skin microvascular perfusion (laser speckle contrast analysis), (ii) fundoscopy (non-mydriatic fundus camera), (iii) indirect assessment of myocardial perfusion (subendocardial viability ratio) and (iv) determination of urine albumin-to-creatinine ratio (UACR). CV risk was calculated using the QResearch Risk Estimator version 3 (QRISK3). Serum Galectin-3 levels were determined. **Results**: Forty-seven SLE patients and fifty controls were studied. SLE patients demonstrated impaired skin microvascular reactivity (160.2 ± 41.0 vs. 203.6 ± 40.1%), retinal arteriolar narrowing (88.1 ± 11.1 vs. 94.6 ± 13.5 μm) and higher UACR levels compared to controls. Furthermore, SLE individuals had significantly higher Galectin-3 levels [21.5(6.1) vs. 6.6(6.6) ng/dL], QRISK3 scores [7.0(8.6) vs. 1.3(3.6)%] and a greater chance for microvascular dysfunction. In the SLE group, patients with multiple TOD exhibited higher QRISK3. In the multivariate analysis, the accumulation of TOD correlated with disease activity and Galectin-3 (*p* < 0.05). **Conclusions**: Our study showed for the first time that SLE patients exhibit a greater number of cases of TOD. The accumulation of TOD was associated with increased CV risk. Clinicians dealing with SLE should be aware and seek microvascular alterations.

## 1. Introduction

Systemic lupus erythematosus (SLE) is a prototype autoimmune disease characterized by a variety of clinical manifestations and severity [1,2]. Despite the marked improvements in SLE therapeutics during the last decades, the disease still has an increased mortality rate compared to the general population [3,4].

Cardiovascular (CV) disease is a well-recognized complication and a major cause of death among SLE individuals [4,5]. Indeed, SLE patients exhibit an increased risk for stroke and myocardial infarction (MI) compared to the general population [5]. Interestingly, the risk of stroke, MI and death seems to have an inverse relationship with age, being higher in younger SLE patients than in older ones [4,5,6]. Although traditional risk factors contribute significantly, they cannot solely explain the increased CV burden that these patients carry [7,8,9,10]. It has been calculated that SLE patients have over a seven-fold higher CV risk than expected based on the traditional risk factors alone [7]. Therefore, several disease-related characteristics (such as the presence of antiphospholipid antibodies) have been identified as important predictors of CV events, and new disease-adapted CV risk prediction tools have been developed for this aim [8,11].

In a recent large population-based study, it was shown that several autoimmune diseases are associated with an increased CV risk. Moreover, the risk increased with the number of autoimmune diseases in each patient. To this end, the authors proposed that autoimmunity per se could be the risk factor for CV disease, having a greater contribution than previously recognized [9]. Inflammation is another recognized contributor to the pathogenesis of CV disease (the inflammation hypothesis of coronary artery disease) and to the progression of both atherosclerosis and arteriosclerosis [9,12]. On the other hand, inhibiting chronic inflammation can lead to significantly lower rates of CV events, independent of any improvement in other risk factors [9,13,14].

During the last decades, subclinical vascular markers have been developed as early indicators of CV disease. The most well-known and well-studied indices are pulse wave velocity (PWV), the gold standard method to assess aortic stiffness and intima–media thickness (IMT), which is a marker of subclinical atherosclerosis [15,16]. Data from a recent meta-analysis showed that patients with SLE exhibit increased arterial stiffness compared to healthy controls, as assessed by PWV [17]. Moreover, SLE patients exhibit accelerated subclinical atherosclerosis, having higher IMT and an increased prevalence of carotid plaques as compared to healthy controls. Indeed, the risk of subclinical atherosclerosis in SLE is comparable to or even higher than in other conditions with increased CV burden [11,18,19,20].

Accumulating evidence suggests that microvascular dysfunction is an important component of the pathophysiology of CV disease, even in the early stages when no clinically detectable complications are observed [21]. Alterations in microcirculation have been documented not only in patients with CV risk factors such as hypertension and diabetes but also in patients with autoimmune rheumatic diseases (ARDs) [22,23,24]. Patients with SLE exhibit coronary microvascular dysfunction, impaired skin microvascular reactivity and blunted cerebral oxygenation compared to controls [25,26,27]. Despite the increasing research interest in the field, the prevalence of microvascular dysfunction in SLE is not precisely known. Furthermore, there is a lack of data regarding the presence of multiple microvascular target organ damage (TOD) in SLE patients and its association with CV risk.

Galectin-3 (Gal-3) is a member of the lectin family and has a high affinity for β-galactosidase [28]. During the past few years, it has emerged as a promising biomarker of CV disease and fibrosis [29,30,31]. Gal-3 is a multifunctional protein involved in a variety of biological processes, such as cell proliferation, differentiation, migration, adhesion and apoptosis [28,32]. On the other hand, Gal-3 also plays an important role in vascular and tissue remodeling and fibrosis [29,30]. Although the underlying mechanisms are not clarified yet, they probably include the JAK/STAT signaling pathway and protein kinase C [33,34]. Moreover, it is involved in the atherosclerotic process through chronic inflammation [31,32]. The predictive role of Gal-3 in patients with heart failure for future CV events, hospitalization and death is well established, and, therefore, it has been introduced in the American Heart Association guidelines to improve risk stratification [32,35]. In addition, its value has been investigated in patients with increased CV risk as well as in the general population [32].

Due to its biological function, Gal-3 could interfere in the development of autoimmunity [36]. Hence, it seems that SLE patients exhibit higher serum Gal-3 levels compared to healthy controls and individuals with other ARDs [37]. Additionally, serum Gal-3 levels correlate with serum anti-double-stranded DNA (anti-dsDNA) antibody titers in individuals with SLE [38]. While serum Gal-3 levels have been associated with macro- and microcirculation indices in patients with rheumatoid arthritis (RA), the role of Gal-3 as a vascular biomarker in SLE remains unclear [39,40].

Therefore, the aims of our study were (i) to non-invasively assess structural and functional microvascular alterations in SLE in various vascular beds and identify their frequency, (ii) to examine possible associations between the accumulation of microvascular TOD and CV risk in patients with SLE and (iii) to compare Gal-3 levels between the groups and investigate whether Gal-3 represents a predictor of combined microvascular TOD.

## 2. Materials and Methods

### 2.1. Participants

Patients who met the Systemic Lupus International Collaborating Clinics (SLICC) classification criteria for SLE were recruited from the Rheumatology Outpatient Unit [41]. The diagnosis of SLE was made by a rheumatology specialist. The control group consisted of individuals matched for age and body mass index (BMI) and cardiovascular disease risk factors, recruited from both the Hypertension Unit of the 3rd Department of Internal Medicine of Aristotle University (Papageorgiou General Hospital, Thessaloniki, Greece) and the community during the same period. None of the participants had a history of established cardiovascular disease. All participants were Caucasian, over 18 years old and gave written informed consent prior to study enrollment [42]. This study was approved by the institutional review board committee and conducted in accordance with the Declaration of Helsinki (2013 revision) [43].

### 2.2. Clinical Assessment

After obtaining a detailed medical history, a thorough physical examination was performed. Activity of the disease was measured using the Systemic Lupus Erythematosus Disease Activity Index 2000 (SLEDAI-2K) score, and permanent organ damage was calculated using the Systemic Lupus International Collaborating Clinics/American College of Rheumatology damage index (SDI) [44,45]. Office blood pressure (office BP) was measured three times in each participant, with 2 min intervals between measurements, according to a standard methodology [46]. The average of the two last measurements was considered the office BP. A validated oscillometric device (Microlife AG, Widnau, Switzerland) with the appropriate cuff size was used. Hypertension was defined as office systolic and/or diastolic BP ≥ 140/90 mmHg and/or current antihypertensive medication.

### 2.3. Laboratory Measurements

Blood samples for laboratory tests were obtained to quantify biochemical profile (levels of uric acid, fasting glucose, creatinine and lipid profile), inflammatory markers (erythrocyte sedimentation rate, C-reactive protein), levels of complement components (C3, C4), antinuclear antibodies and anti-double-stranded DNA antibodies. Antiphospholipid antibody positivity (lupus anticoagulant, anticardiolipin antibody, antibody to β2 glycoprotein I) was retrieved from patients’ medical history file. Glomerular filtration rate was estimated in mL/min/1.73 m^2^ using the chronic kidney disease epidemiology collaboration equation [47]. Moreover, serum from blood samples was separated and stored at −80 °C for Galectin-3 level detection and quantification, as previously described [39]. A commercially available competitive enzyme-linked immunosorbent assay kit (ELISA kit) for Galectin-3 [Catalog No. AMS.E0497h, AMS Biotechnology (AMSBIO Europe) Ltd., Alkmaar, The Netherlands], with a detection range of 2.5–160.0 ng/dL, was used in the present study. All samples were analyzed in duplicates by the same investigator, and results are shown in ng/dL.

### 2.4. Cardiovascular Risk Assessment

CV risk was calculated using the QResearch Risk Estimator version 3 (QRISK3) [42]. The score estimates the risk of developing CV disease over the next 10 years and includes SLE as an independent CV risk factor. Information is applicable to ages 25–84.

### 2.5. Microcirculation Assessment

**Figure 1:**
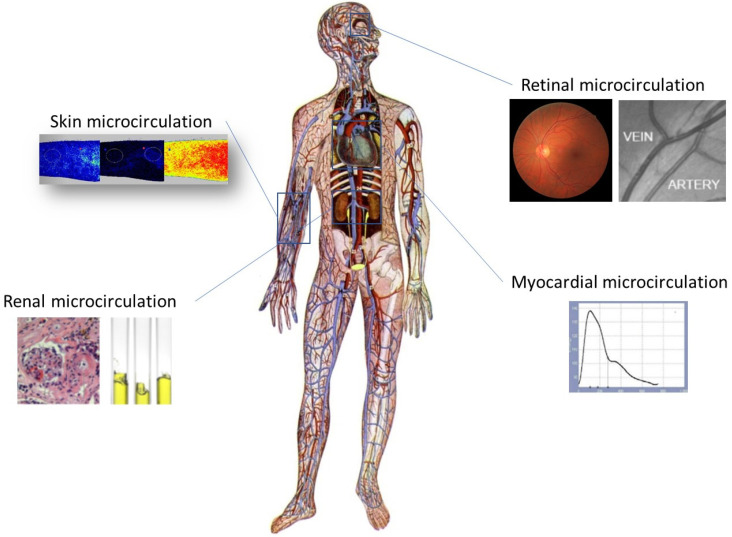
Easily accessible microvascular beds used for the study of microcirculation. Modified by Triantafyllou et al. [48].

Participants arrived at the laboratory in the morning hours after an overnight fast. All measurements were performed in a separate, quiet and temperature-controlled room. They were instructed to abstain from smoking and drinking coffee, tea or alcohol for 4 h before testing. All microvascular beds assessed in the present study are summarized in Figure 1.

#### 2.5.1. Assessment of Skin Microvascular Function

Evaluation of skin microvascular perfusion was performed using laser speckle contrast analysis (LASCA) coupled with the post-occlusive reactive hyperemia (PORH) protocol, as previously described [23,25]. LASCA is a relatively new, non-invasive method to evaluate skin microvascular perfusion in real time and with high reproducibility [49,50,51,52]. A LASCA device (PeriCam PSI NR System, Perimed, Järfälla, Sweden) with a laser wavelength of 785 mm was used. Briefly, after a 20 min acclimatization period, a 3 min baseline period was recorded. Then, a pressure cuff was inflated at suprasystolic levels (250 mmHg) to obstruct blood flow in the brachial artery for 5 min (occlusion period), and thereafter the cuff was rapidly deflated and a 5 min post-occlusive recording period followed. Data were analyzed using the manufacturer’s software. Two circular skin sites (10 mm radius) were randomly chosen on the ventral surface of the forearm, and the average blood perfusion of the two areas was used in the analysis. Areas with visible veins, hair growth, tattoos, skin pigmentation or other scars were avoided during the measurement. Recorded values are expressed in arbitrary perfusion units (PUs). Using data from measurements performed at the Hypertension Unit of the 3rd Department of Internal Medicine, in a population of otherwise healthy volunteers, microvascular reactivity ≤ 5th percentile was considered abnormal. To date, there is no consensus regarding normal values for skin microvascular reactivity; however, reference values generally include 95% of the observations [53].

#### 2.5.2. Retinal Vessel Analysis

Participants underwent bilateral fundoscopy and fundus photography using a non-mydriatic fundus camera (NIDEK AFC-230/210, NIDEK, Fremont, CA, USA). Two photographs from each eye were obtained, and the ones with the best quality were used in the analysis. Consequently, the images were analyzed using specifically designed semiautomated computer software, as described elsewhere [54,55]. Two trained authors (AT and AS), blind to the participants’ identities, independently conducted the analysis, and in cases of disagreement, a consensus was reached after discussion. Central retinal artery equivalent (CRAE) and central retinal vein equivalent (CRVE) were automatically calculated using the modified Parr and Hubbard formula [56]. Retinal arteriovenous ratio (AVR) was calculated as the CRAE/CRVE ratio. Using data from measurements performed at the Hypertension Unit of the 3rd Department of Internal Medicine, in a population of otherwise healthy volunteers, CRAE values ≤ 5th percentile were considered abnormal. To our knowledge, there is no consensus regarding reference values for CRAE.

#### 2.5.3. Assessment of Microvascular Myocardial Perfusion

Subendocardial viability ratio (SEVR) was used as an indirect index of myocardial perfusion. SEVR, also known as the Buckberg index, reflects the balance between oxygen supply and demand [57,58]. It is calculated as the ratio of the area under the central aortic pressure waveform during diastole (oxygen supply) to the area under the central aortic pressure waveform during systole (oxygen needs) [57,58,59]. SEVR correlates with invasive measurements of coronary flow reserve, and it can be used as a tool for indirect assessment of myocardial perfusion [57,60]. SEVR was estimated via applanation tonometry of the radial artery using the SphygmoCor device (AtCor Medical, West Ryde, NSW, Australia). Measurements were performed in the supine position after a 15 min rest period, according to a predetermined protocol, and the average of two consecutive measurements was used in the analysis [15]. Reference values of central hemodynamic parameters from a European population, controlled for age and sex, were used in the present study [61].

#### 2.5.4. Assessment of Urinary Albumin Excretion

Urinary albumin excretion was used as an indirect index of renal microcirculation, as it reflects not only renal endothelial dysfunction but also more generalized vascular damage [62,63,64,65]. Urinary albumin excretion was determined by urine albumin-to-creatinine ratio (UACR) in a random urine sample (Afinion ACR, Abbott, IL, USA). Although 24 h urine collection is considered the gold standard for the estimation of urinary albumin excretion, UACR is a convenient, reliable and comparable method [66,67]. Increased albumin excretion was defined as UACR ≥ 30 mg/g [67].

### 2.6. Statistical Analysis 

Statistical analyses were performed using SPSS software (IBM SPSS Statistics 25.0, Chicago, IL, USA). Normally distributed continuous variables are described as mean ± standard deviation, while non-normally distributed variables are described as median ± interquartile range, based on the normality of the distribution. Differences among groups were examined by independent sample *t*-tests or one-way ANOVA for normally distributed variables, whereas the non-parametric Mann–Whitney or Kruskall–Wallis test was used for non-normally distributed variables. Qualitative variables were compared by the χ^2^ test or Fisher’s exact test when necessary, and results are expressed as percentages. Pearson’s or Spearman’s correlation coefficient was used based on the variable’s normality of distribution. Odds Ratio (OR) for microvascular dysfunction was calculated. Furthermore, in order to explore possible associations between risk factors and the number of microcirculation TOD cases, a multivariate regression analysis was applied. A *p* value < 0.05 was considered statistically significant.

## 3. Results

### 3.1. Participants’ Characteristics

In total, 97 individuals (47 SLE patients and 50 controls) aged 46.7 ± 10.1 years were included in this study. The baseline characteristics of the study participants are presented in Table 1. No statistically significant differences between the groups were observed in age, sex, BMI, office BP or smoking status. Patients with SLE presented increased levels of circulating Galectin-3 [21.5(6.1) vs. 6.6(6.6) ng/dL, respectively, *p* < 0.001] and a higher estimated 10-year CV risk [7.0(8.6) vs. 1.3(3.6)%, respectively, *p* < 0.001] compared to controls. 

Participants in the SLE group (Table 2) had a median disease duration of 12.0 (5.0–18.0) years. As expected, 87.2% of the SLE patients were women. Two-thirds (66%) of the patients were on antimalarial treatment, whereas less than half of them (46.8%) were treated with corticosteroids [median dose 5.0 (7.5) mg of prednisolone equivalent] and 44.7% were under treatment with immunosuppressants (mainly azathioprine, 29.8%). The majority of the patients (91.5%) were antinuclear antibody (ANA) positive, and 45.7% had positive anti-dsDNA antibodies (Table 2).

### 3.2. Vascular Measurements

Table 3 summarizes the microvascular assessment in all vascular beds. Regarding skin microcirculation, baseline perfusion was significantly higher in SLE patients compared to controls, while peak perfusion and the percentage decrease in perfusion during arterial occlusion did not differ significantly between the two groups. During reperfusion, peak flux increased in both groups; however, patients with SLE exhibited a blunted peak magnitude compared to controls (160.2 ± 41.0 vs. 203.6 ± 40.1%, respectively, *p* < 0.001). Moreover, individuals in the SLE group presented lower retinal artery diameter compared to the control group (CRAE 88.1 ± 11.1 vs. 94.6 ± 13.5 μm, respectively, *p* < 0.05), whereas no statistically significant differences were observed in CRVE or AVR. Regarding urinary albumin excretion, patients with SLE exhibited significantly higher UACR levels compared to controls [8.9(16.2) vs. 5.7(2.6) mg/g, respectively, *p* < 0.05]; however, the results did not remain statistically significant after excluding individuals with a known history of lupus nephritis [6.3(8.1) vs. 5.7(2.6)]. In addition, SEVR did not differ between the two groups either. Among patients with SLE, only UACR showed a positive association with QRISK3 (r = 0.474, *p* = 0.006) and Galectin-3 levels (r = 0.414, *p* = 0.012).

### 3.3. Prevalence of Microvascular Target Organ Damage

An attempt to investigate the prevalence of microvascular alterations in SLE was performed. Two out of three SLE patients exhibited microvascular dysfunction of at least one target organ compared to the control group (63.8% vs. 14%, respectively, *p* < 0.001). This can be translated into a greater chance of microvascular dysfunction in SLE patients compared to controls (OR: 10.8, 95% CI: 4.0–29.4). When tested separately, individuals with SLE demonstrated higher rates of dysfunction in each vascular bed as compared to controls; however, only skin microvascular reactivity (23.4% vs. 0%, *p* = 0.002) and albuminuria (22.2% vs. 0%, *p* = 0.026) were statistically significant.

### 3.4. Associations of Combined Microvascular Target Organ Damage

**Figure 2:**
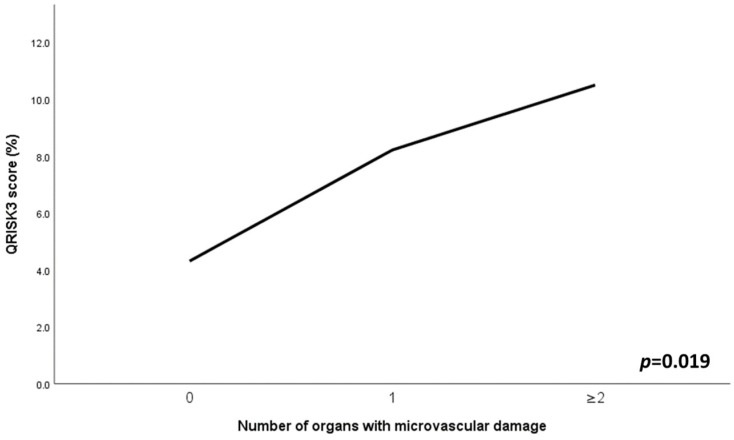
Cardiovascular risk according to microvascular target organ damage. QRISK3: QResearch Risk Estimator version 3.

In the SLE group, individuals with multiple (at least two different vascular beds) TOD exhibited a higher QRISK3 score (*p* < 0.05), as depicted in Figure 2. In addition, accumulation of microvascular TOD was positively correlated with QRISK3 score (r = 0.440, *p* = 0.004), systolic BP (r = 0.301, *p* = 0.040) and Galectin-3 levels (r = 0.343, *p* = 0.018), while a trend for SLEDAI-2K was observed (*p* = 0.062). In the multivariate analysis, only SLEDAI-2K (β = 0.343, *p* = 0.020) and Galectin-3 levels (β = 0.350, *p* = 0.018) remained independent predictors of multiple TOD.

## 4. Discussion

To our knowledge, this is the first study to investigate the concomitant presence of microvascular TOD, both structural and functional, in different vascular beds among SLE patients. The results revealed that patients with SLE demonstrate impaired skin microvascular reactivity, retinal arteriolar narrowing and higher urinary albumin excretion as compared to individuals matched for age, BMI, sex and BP levels. SEVR was lower in the SLE group; however, it did not reach statistical significance. By study design, the two groups were matched for age, sex and BMI, minimizing the possible confounding effect of those parameters. Taking into account that BP and smoking status may affect microvascular function, groups with similar BP levels and smoking statuses were included in this study.

Microvascular dysfunction has been individually assessed in SLE patients in previous studies. In a study by our team, it was shown that patients with SLE exhibit blunted skin microvascular reactivity compared to controls, independent of CV disease or risk factors [25]. Increased baseline perfusion has been observed both in patients with SLE and early systemic sclerosis. A proposed hypothesis for this early microvascular impairment was that during baseline, more functional vessels are recruited, but this is not enough to compensate for the ischemic stimulus, leading to significantly lower microvascular reactivity during reperfusion. Regarding retinal microcirculation, Lee et al. also found narrower retinal arteries in SLE patients compared to healthy controls, although the results did not reach statistical significance [68]. In a large study of the Greek population, retinal arterial diameter was comparable between individuals with ARDs (including 75 patients with SLE) and the control group. However, the majority of the participants in the control group had hypertension, and there was a statistically significant difference in the baseline characteristics of the two groups that could have interfered with the results [69]. In accordance with our findings, previous studies have shown that individuals with RA have decreased retinal arteriolar diameter compared to controls [70]. Patients with RA also show lower SEVR values as compared to controls, a finding that was not confirmed in the present study, probably due to the small sample size.

Moving one step ahead, our study is the first to thoroughly assess the burden of microvascular TOD in SLE patients by using a variety of non-invasive techniques to assess microvascular structure and function. Remarkably, most of the SLE patients (63.8%) exhibited microvascular dysfunction in one or more target organs compared to controls. This further indicated that patients with SLE display a greater chance of microvascular dysfunction. In addition, among the examined vascular beds, the skin and kidneys appeared to be the ones most affected. Taking into consideration that urinary albumin excretion is affected by lupus nephritis history, our consistent finding of impaired skin microvascular reactivity strongly highlights the importance of skin microcirculation as a more reliable tool for the early detection of generalized microvascular damage in SLE patients.

Another interesting finding is that, among individuals with SLE, an increase in the number of microvascular TOD cases is correlated with estimated CV risk. In fact, SLE patients with multiple TOD exhibited a higher QRISK3 score. In the present study, the QRISK3 score was used to calculate CV risk. QRISK3 is an SLE-adapted score to estimate the risk of developing CV disease over the next 10 years. It has better performance in predicting the risk of CV disease as compared to generic CV risk calculators [71]. Furthermore, QRISK3 correlates with IMT and can help discriminate the presence of carotid plaque and predict plaque progression [11,72,73]. It is also associated with indices of aortic stiffness and endothelial dysfunction [72,74]. As such, our finding of a significant association between the clustering of microvascular TOD and CV risk in SLE patients strongly indicates the importance of the early identification of microvascular dysfunction in SLE patients through easily accessible vascular beds, a practice that could alert clinicians in advance and increase their CV risk awareness.

The underlying mechanisms of impaired microvascular function in SLE remain uncertain, and several factors have been implicated in the development of both functional and structural alterations. Circulating autoantibodies in SLE patients that can form immune complexes may be deposited in the vessels, resulting in endothelial damage and increased vascular permeability. Furthermore, anti-endothelial cell antibodies are often present and may contribute to microvascular dysfunction by activating endothelial cells and promoting the expression of adhesion molecules, the migration of immune cells and complement activation [25,75]. In the present study, it was shown for the first time that Gal-3 levels correlate significantly with the number of affected target organs. Indeed, Gal-3 remained a significant predictor of multiple TOD even after adjustment for other factors. Our results suggest that Gal-3 may be involved in the development of microvascular dysfunction in SLE. Available studies evaluating the role of Gal-3 in SLE mainly address its involvement in the pathogenesis, clinical manifestations and activity of the disease. In agreement with our results, Gal-3 has already been used as a marker not only of disease activity and severity but also of cardiac function in patients with RA [39,40].

Gal-3 is a multifunctional protein involved in a variety of biological processes; however, its contribution to the pathogenesis of vascular damage has not been clarified yet. Gal-3 is involved in vascular damage through macrophage chemoattraction and activation [76,77]. Thus, Gal-3-expressing macrophages have been associated with the abnormal elimination of microvasculature in experimental models [78]. Additionally, Gal-3 can mediate neutrophil adhesion and recruitment. Another possible pathway includes the promotion of reactive oxygen species generation [77]. More recent data, however, suggest that Gal-3 may be involved in complement activation through the C1q component [79]. It would be interesting for future studies to explore the possible interaction of Gal-3 with C1q (especially in patients with SLE) and its receptor, which is an emerging molecule in CV disease [80].

Despite the interesting, novel and first-reported findings of our study, there are some unavoidable limitations. Firstly, the relatively small sample size of this study can be partly explained by the epidemiology of the disease itself. Additionally, all the participants were of European ancestry, making it difficult to generalize the results to other populations. Another limitation of this study is that the prevalence of microvascular TOD was influenced by the cutoff values used, as there is no consensus regarding normal values for skin and retinal microcirculation. This also limits their widespread use in everyday clinical practice. In most cases, reference values include 95% of the observations, a rule that was used in the present study as well [53].

In conclusion, our novel data show that patients with SLE exhibit skin, renal and retinal microvascular dysfunction as compared to controls. It appears that patients with SLE have a greater chance of having microvascular dysfunction compared to controls. A significantly greater percentage of SLE individuals demonstrate alterations in microcirculation affecting one or more target organs compared to the control group. The accumulation of microvascular TOD was associated with an increased CV risk profile. This finding may partially explain the increased CV risk of these patients and underlies the significance of examining microcirculation in patients with SLE using easily accessible windows, such as the skin or the retina. Gal-3 levels correlated with the number of affected target organs and predicted the accumulation of TOD independently, highlighting its contribution not only to the development of the disease itself but also to its complications. However, larger studies are needed to confirm the results and to explore additional prognostic factors and associations with specific manifestations and other micro- or macrocirculation indices.

## Figures and Tables

**Table 1:** Baseline characteristics of the study population.

	SLE (*n* = 47)	Control (*n* = 50)	*p* Value
Age (years), mean ± S.D.	48.5 ± 9.2	45.0 ± 10.7	0.092
BMI (Kg/m^2^), mean ± S.D.	25.5 ± 4.4	26.4 ± 4.8	0.337
Female sex, n (%)	41 (87.2)	38 (76.0)	0.155
Smoking, yes, n (%)	23 (48.9)	17 (34.0)	0.135
Office SBP (mmHg), mean ± S.D.	118.9 ± 14.1	118.8 ± 13.2	0.991
Office DBP (mmHg), mean ± S.D.	77.3 ± 11.6	75.6 ± 8.5	0.406
Office HR (pulses/min), median (IQR)	73.0 (16.0)	75.0 (15.0)	0.411
Glucose (mg/dL), mean ± S.D.	85.9 ± 8.9	88.7 ± 8.8	0.152
Uric acid (mg/dL), mean ± S.D.	4.6 ± 1.0	4.8 ± 1.1	0.501
eGFR (mL/min/1.73 m^2^), median (IQR)	93.0 (24.0)	96.0 (17.0)	0.200
Total Cholesterol (mg/dL), mean ± S.D.	177.4 ± 28.9	191.4 ± 35.5	0.041
Triglycerides (mg/dL), median (IQR)	84.0 (60.0)	87.0 (53.0)	0.622
HDL Cholesterol (mg/dL), mean ± S.D.	50.5 ± 15.5	50.6 ± 9.1	0.981
LDL Cholesterol (mg/dL), mean ± S.D.	107.0 ± 24.4	117.8 ± 30.7	0.066
Galectin-3 (ng/dL), median (IQR)	21.5 (6.1)	6.6 (6.6)	<0.001
QRISK3 score, median (IQR)	7.0 (8.6)	1.3 (3.6)	<0.001

SLE: Systemic lupus erythematosus; S.D.: standard deviation; IQR: interquartile range; BMI: body mass index; SBP: systolic blood pressure; DBP: diastolic blood pressure; HR: heart rate; eGFR: estimated glomerular filtration rate; HDL: high-density lipoprotein; LDL: low-density lipoprotein; QRISK3: QResearch Risk Estimator version 3.

**Table 2:** Characteristics of patients with SLE (*n* = 47).

Clinical Characteristics	
Age (years), mean ± S.D.	48.5 ± 9.2
Disease duration (years), median (IQR)	12.0 (13.0)
Female sex, *n* (%)	41 (87.2)
Raynaud’s phenomenon, *n* (%)	24 (54.5)
Lupus nephritis history, *n* (%)	8 (18.6)
SLEDAI-2K, median (IQR)	2.0 (2.0)
SDI, median (IQR)	0.6 (1.0)
Serology	
ANA, positive, (%)	91.5
Anti-dsDNA, (%)	45.7
aPL positivity (%)	32.6
ESR (mm), median (IQR)	13.0 (19.0)
CRP (mg/dL), median (IQR)	0.26 (0.47)
C3, mean ± S.D.	75.2 ± 20.2
C4, mean ± S.D.	14.0 ± 5.6
Treatment	
Hydroxychloroquine, yes (%)	66.0
Corticosteroid use, yes (%)	46.8
Immunosuppressants, yes (%)	44.7
Azathioprine (%)	29.8
Mycophenolate mofetil (%)	6.4
Cyclophosphamide (%)	4.3
Methotrexate (%)	4.3
Belimumab (%)	2.1

SLE: Systemic lupus erythematosus; S.D.: standard deviation; IQR: interquartile range; SLEDAI-2K: Systemic Lupus Erythematosus Disease Activity Index 2000; SDI: Systemic Lupus International Collaborating Clinics/American College of Rheumatology damage index; ANA: antinuclear antibody; anti-dsDNA: anti-double-stranded DNA antibody; aPL: antiphospholipid antibody; ESR: erythrocyte sedimentation rate; CRP: C-reactive protein; C3: complement component 3; C4: complement component 4.

**Table 3:** Microvascular assessment of the study population.

	SLE (*n* = 47)	Control (*n* = 50)	*p* Value
Baseline flux (PU), mean ± S.D.	43.4 ± 7.8	38.4 ± 9.9	0.012
Baseline-to-occlusion change (%), median (IQR)	−79.0 (11.5)	−79.0 (12.4)	0.580
Peak flux (PU), mean ± S.D.	112.0 ± 23.2	114.7 ± 26.6	0.627
Peak magnitude (%), mean ± S.D.	160.2 ± 41.0	203.6 ± 40.1	<0.001
CRAE (μm), mean ± S.D.	88.1 ± 11.1	94.6 ± 13.5	0.022
CRVE (μm), mean ± S.D.	116.1 ± 14.0	117.5 ± 15.4	0.664
AVR, median (IQR)	0.78 (0.18)	0.78 (0.15)	0.346
SEVR (%), mean ± S.D.	150.2 ± 20.7	154.1 ± 28.8	0.493
UACR (mg/g), median (IQR)	8.9 (16.2)	5.7 (2.6)	0.041

SLE: Systemic lupus erythematosus; PU: perfusion unit; S.D.: standard deviation; IQR: interquartile range; CRAE: central retinal artery equivalent; CRVE: central retinal vein equivalent; AVR: arteriovenous ratio; SEVR: subendocardial viability ratio; UACR: urine albumin-to-creatinine ratio.

## Data Availability

Data are available upon request from the corresponding author.
